# Low-Frequency Noise Investigation of 1.09 μm GaAsBi Laser Diodes

**DOI:** 10.3390/ma12040673

**Published:** 2019-02-24

**Authors:** Justinas Glemža, Vilius Palenskis, Andrejus Geižutis, Bronislovas Čechavičius, Renata Butkutė, Sandra Pralgauskaitė, Jonas Matukas

**Affiliations:** 1 Institute of Applied Electrodynamics and Telecommunications, Vilnius University, Saulėtekio av. 3, LT-10257 Vilnius, Lithuania; vilius.palenskis@ff.vu.lt (V.P.); sandra.pralgauskaite@ff.vu.lt (S.P.); jonas.matukas@ff.vu.lt (J.M.); 2 Department of Optoelectronics, Center for Physical Sciences and Technology, Saulėtekio av. 3, LT-10257 Vilnius, Lithuania; andrejus.geizutis@ftmc.lt (A.G.); bronislovas.cechavicius@ftmc.lt (B.Č.); renata.butkute@ftmc.lt (R.B.)

**Keywords:** current-voltage characteristics, defects, electrical noise, fluctuations, GaAsBi laser diode, leakage channel

## Abstract

GaAsBi is a suitable and very attractive material system to be used as an active layer in laser diodes (LDs). To understand the performance and the reliability of such devices and also for further laser diode improvements, the origin of noise sources should be clarified. A detailed study of near-infrared 1.09 μm wavelength GaAsBi type-I laser diodes using the low-frequency noise spectroscopy in a temperature range of (180–300) K is presented. Different types of voltage fluctuation spectral density dependencies on the forward current far below the lasing threshold have been observed. According to this, investigated samples have been classified into two groups and two equivalent noise circuits with the corresponding voltage noise sources are presented. Calculations on the voltage spectral density of the electrical noise and current-voltage characteristic approximations have been performed and the results are consistent with the experimental data. The analysis showed that one group of LDs is characterized by 1/*f*^α^-type electrical fluctuations with one steep electrical bump in the electrical noise dependence on forward current, and the origin of these fluctuations is the surface leakage channel. The LDs of the other group have two bumps in the electrical noise dependence on current where the first bump is determined by overall LD defectiveness and the second bump by Bi-related defects in the active area of LD with characteristic Lorentzian-type fluctuations having the activation energy of (0.16–0.18) eV.

## 1. Introduction

Bismuth containing GaAs (GaAs_1-*x*_Bi*_x_*) is a novel and very promising material system for optoelectronic devices, especially in fiber optic communication laser diodes (LDs) operating in the (1.3–1.5) μm wavelength region due to numerous advantages [1,2]. Firstly, the incorporation of a small amount of Bi atoms into GaAs leads to a strong band gap energy reduction by (84–91) meV/%Bi [3], as well as temperature coefficient of the band gap being less sensitive to temperature [3,4]. Moreover, for relatively high Bi composition (*x* > 10%), the spin-orbit splitting energy (Δ_so_) becomes larger than the band gap, which lets us expect the suppression of Auger recombination and inter-valence band absorption processes [1,3]. This factor is the most important for bismide alloy application as active layers in telecommunication laser diodes [1]. Considering future applications, GaAsBi material is a potential candidate to be used in bio-inspired stretchable optoelectronics systems, e.g., for medicine purposes, military or industry devices [5,6,7]. Despite the brittle and non-stretchable nature of GaAsBi, flexible electronic devices using the other brittle inorganic semiconductors such as GaAs or Si have already been successfully demonstrated [5].

However, the fast and wide application of GaAsBi alloys and the emission wavelength extension is slowed down by technological difficulties related to the growth of this material system. The growth temperature has to be lowered to ≤400 °C (the optimum growth temperature of GaAs is ~ 590 °C [8]) in order to prevent Bi surface segregation [1,2,3,4]. Due to this, it is difficult to produce laser structures with more than 4.4% of Bi by metal-organic vapor phase epitaxy (MOVPE). To incorporate more than 10% of Bi, molecular-beam epitaxy (MBE) is usually used, where lower growth temperature can be applied [1].

Surface segregation is not the only issue making the growth quality of this material worse, e.g., low As flux leads to the surface roughness, while excess of Bi forms droplets on the surface [2,4,9]. There are several methods allowing us to suppress these effects. It has been demonstrated that the two-substrate-temperature MBE technique, where GaAsBi quantum wells (QWs) are grown at lower temperature and GaAs layers at higher, significantly reduces the Bi segregation [2]. Also, a hybrid approach has been presented, where the active region is grown by MBE and the rest of the LD structure by MOVPE with the all following MOVPE advantages [1,10].

Despite all the mentioned improvements in GaAsBi growth methods, crystal quality still depends on a variety of factors and processes such as non-radiative recombination, non-uniform distribution of charge carriers, and carrier localization have a huge impact on electrical and optical characteristics of the alloy and overall semiconductor laser [1,4,8]. Thus, it is important to understand physical processes occurring not only during the growth of a laser diode but also during the device operation that affect LD’s performance and reliability.

Due to the sensitivity to various defects, imperfections and structural non-idealities, low-frequency noise characteristics of optoelectronic devices are very informative. While 1/*f*-type electrical fluctuations give information on the general quality of a material or a device, the generation-recombination (g-r) noise helps to detect and identify local energy levels in a semiconductor [11,12,13]. In [14,15,16] it has been demonstrated that the low-frequency noise spectroscopy is a suitable, very effective and non-destructive characterization method for LDs as the measurements are performed at a near equilibrium state. What is more, the investigation of samples is possible at bias currents far below the threshold current of laser generation [16,17]. Nevertheless, the obtained noise characteristics contain information about physical processes that more or less influence semiconductor laser operation at lasing and overall device reliability. The benefit of such a testing method is very important especially for multimode Fabry–Perot LDs—the sample is not only protected from damage of its internal structure caused by high flowing current through the specimen, but also the noise component, generated due to the mode hopping effect, is not recorded as the LD operates in the subthreshold region. Usually the level of noise amplitude during mode-hopping increases drastically and overheads the fluctuations from other noise sources [15,18], aggravating analysis. The noise behavior in a GaAsBi material system is not sufficiently studied as we have not found any GaAsBi LDs research based on low-frequency noise spectroscopy.

In this paper, we report a comprehensive investigation of 1.09 μm type-I GaAsBi laser diodes through the low-frequency noise characteristics at forward bias. The aim of this work is to determine the noise sources, to identify their possible origin and to propose equivalent circuits of the investigated devices based on LDs current-voltage and electrical noise characteristics.

## 2. Fabrication of Laser Diodes and Noise Measuring Technique

### 2.1. Investigated Laser Diodes Structure, Growth and Optical Characteristics

Multiple GaAsBi/GaAs quantum well-based laser diodes were grown using molecular beam epitaxy (MBE) reactor SVT-A equipped by standard metallic Ga, Al and Bi cells, and unique design source for arsenic containing two independently controlled thermal zones for the bulk evaporator and cracking head for generating pure As_2_ flux. The laser structure was grown on an *n*-type GaAs substrate (doped by Si; carrier density about 2 × 10^18^ cm^−3^) oriented in the (100) crystalline plane.

The 1.4 μm-thick AlGaAs cladding layers containing 30% of Al and doped by Si and Be served as waveguides as well *n*- and *p*-contact layers in the laser structure, respectively. The AlGaAs layer was deposited on a 600 °C temperature substrate. The active area of a laser diode consisted of five 8 nm-thick GaAsBi wells (with 8% of Bi) surrounded by GaAs barriers. During the growth of a quantum well structure, the temperature was reduced to 360 °C. The bismide quantum wells were grown supplying arsenic and gallium fluxes with the beam equivalent pressure (BEP) ratio close to 1.09. The Bi to As BEP ratio was set to about 0.4 during the growth of QWs. To compensate residual *n*-type conductivity of pristine GaAs, the GaAs barriers were doped by Be. The growth temperature of barriers was 450 °C. The width of the outer and inner barriers was 25 nm and 7 nm, respectively. The observed reflection high-energy electron diffraction (RHEED) images during the growth of GaAsBi QW—(2 × 1) and GaAs barriers—(2 × 4) demonstrated stoichiometric BEP ratios of group V and III materials.

Finally, the temperature was increased to 600 °C, and 250 nm of highly Be-doped GaAs were grown on the top of the structures to improve the metal-semiconductor contact.

**Figure 1:**
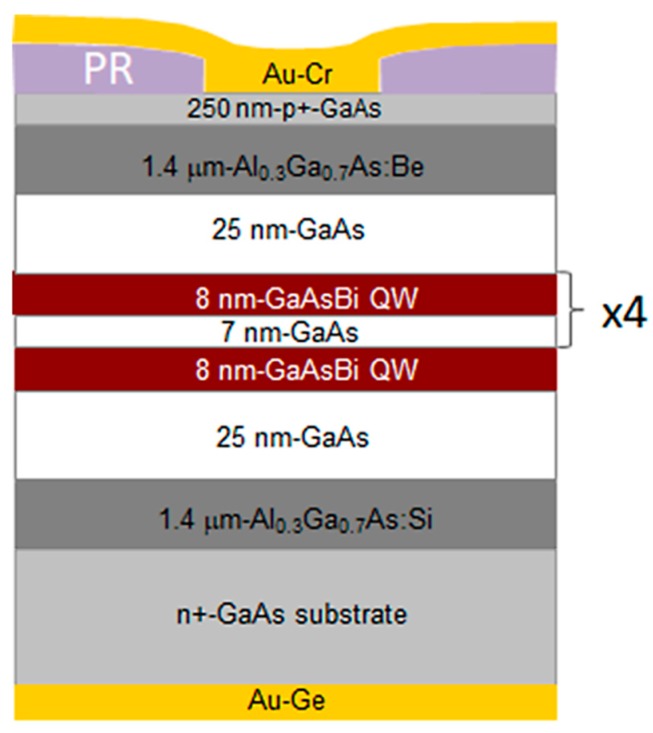
A schematic laser diode structure with 5 GaAsBi quantum wells (QWs).

The 20 μm wide and (2–3) mm long Fabry–Perot cavities were formed by the cutting and leaving the facets as-cleaved. A 200 nm-thick Au-Ge contact was deposited on the bottom of thinned (to ~200 μm) substrate, and Au/Cr metal bilayer with thickness of about 250 nm was deposited through an open photoresist (PR) layer on the top of the epitaxial structure. The laser diodes were annealed inside a rapid thermal annealing (RTA) oven at 400 °C for 180 s to improve the metal-semiconductor contact. The sketch of the LD structure with 5 GaAsBi QWs is presented in Figure 1.

**Figure 2:**
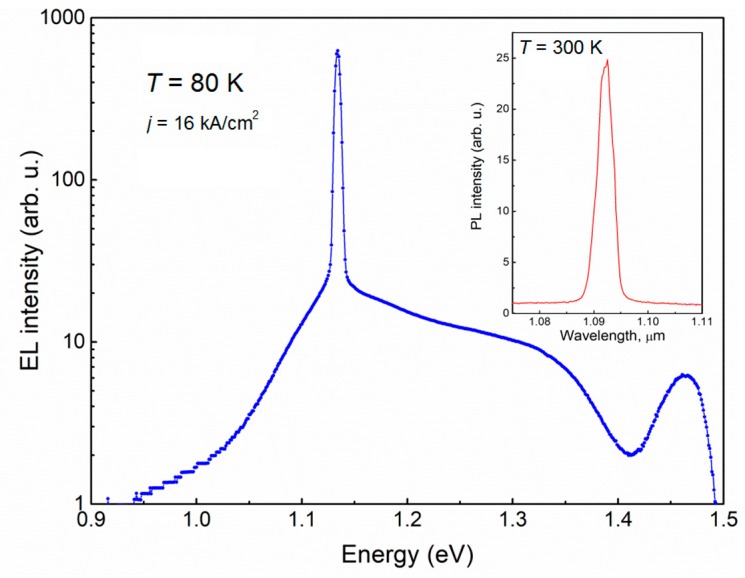
Electroluminescence spectrum measured for the laser diode at the temperature of 80 K. The room temperature (RT) dependence of photoluminescence (PL) intensity on wavelength is presented in the inset.

An optical spectrum analyzer and optical power meters were used for the facet emission and pure spontaneous emission measurements of the as-cleaved LDs. Figure 2 demonstrates electroluminescence (EL) spectrum of the laser diode. The maximum EL peak was reached at 1.13 eV (~1.10 μm) when the temperature was 80 K. The radiation of spontaneous emission was collected from the opening on the top *p*-side stripe contact. The typical photoluminescence (PL) spectrum measured at room temperature (RT) with central peak at ~1.09 μm corresponding to ~8% of Bi in the well region is shown in the inset of Figure 2.

### 2.2. Details on Noise Measurement Technique

**Figure 3:**
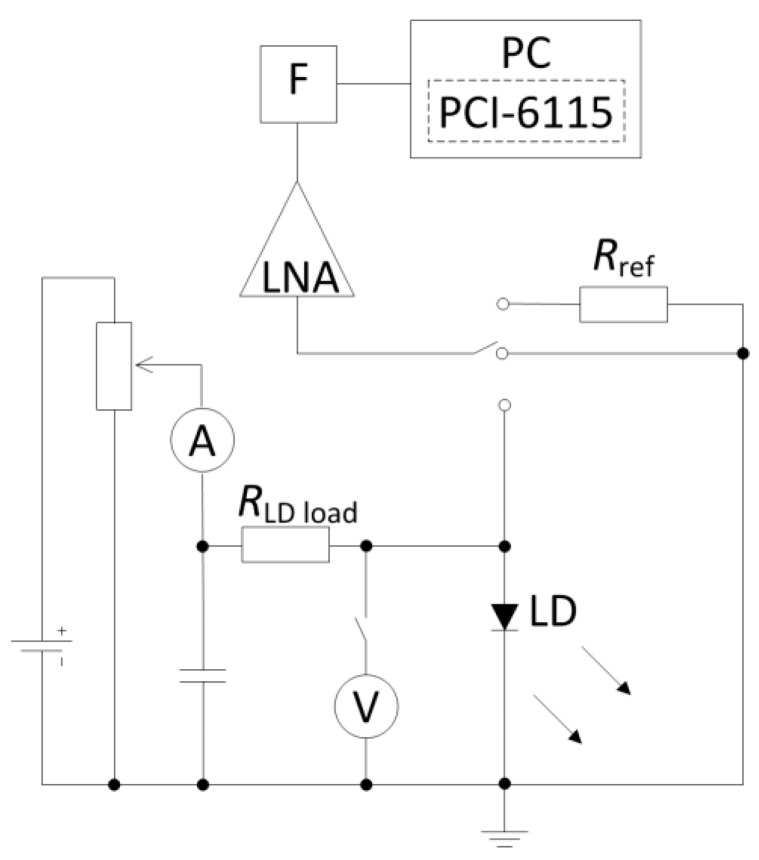
The noise measurement system: LD is the laser diode under investigation, *R*_LD load_ is a load resistor, *R*_ref_ is a reference resistor, LNA is a low-noise amplifier, F is a filter system, PC is a personal computer with PCI-6115 analogue-to-digital converter and fast Fourier transform (FFT)-based spectrum analyzer program.

The low-frequency 10 Hz–200 kHz electrical noise (laser diode terminal voltage fluctuations) have been measured under constant forward bias current operation at RT using a thermoelectric cooler for stabilization of sample temperature and liquid nitrogen cooling for measurements in a (180–300) K temperature interval. The current–voltage (*I*–*U*) and noise characteristics have been measured in a current range far below threshold (up to 100 mA) where the temperature stabilization was valid in order to suppress the self-heating effect and to avoid damage of the quantum well region. Due to this reason, the lasing in these samples is achieved by a pulsed current operation. The noise measurement circuit has a low-noise amplifier, a filter system, an analogue-to-digital converter and a personal computer (Figure 3). The current generator mode is guaranteed for the LDs by choosing appropriate bias voltage and the load resistance *R*_LD load_, that is at least ten times higher than differential resistance of sample under investigation in a whole current range. A specially designed low-noise amplifier (LNA in Figure 3) with a noise equivalent resistance of 13 Ω (at 1 kHz) has been used to amplify the fluctuations. After amplification stage, this analogue signal was converted to a digital one by an analogue-to-digital converter (PCI-6115, National Instruments, Austin, Texas, United States). The spectral density of voltage fluctuations is estimated by the Cooley–Tukey fast Fourier transform-based spectrum analyzer program. The absolute spectral density value of measured noise voltage fluctuation is calculated by comparing it with the thermal noise of reference resistance, *R*_ref_:

(1) $$S_{U}=\frac{\bar{\Delta u_{\mathrm{LD}}^{2}}-\bar{\Delta u_{\mathrm{syst}}^{2}}}{\bar{\Delta u_{\mathrm{ref}}^{2}}-\bar{\Delta u_{\mathrm{syst}}^{2}}}4kT_{0}R_{\mathrm{ref}};$$

where $\bar{\Delta u_{\mathrm{LD}}^{2}}$, $\bar{\Delta u_{\mathrm{syst}}^{2}}$ and $\bar{\Delta u_{\mathrm{ref}}^{2}}$ are, respectively, the variances of the laser diode, the measuring system, and the reference resistor thermal noises in a narrow frequency band Δ*f*; *T*_0_ is the absolute temperature of the reference resistor. The noise measurements have been performed in a laboratory screened by iron and copper sheets—the Faraday cage. For *I*–*U* characteristic’s evaluation, a precision source/measure unit Keysight B2901 has been used.

## 3. Results and Discussion

### 3.1. Electrical Noise Characteristics of GaAsBi Laser Diodes

**Figure 4:**
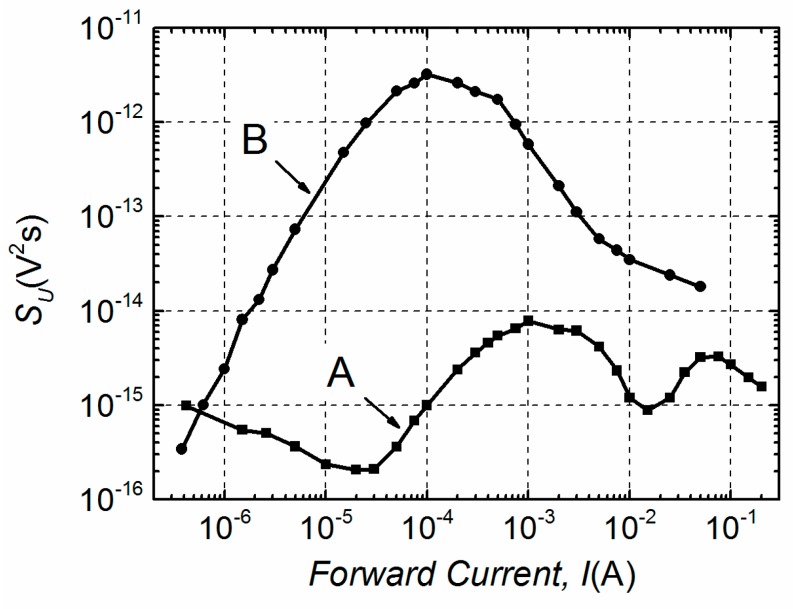
The experimental voltage fluctuation spectral density (*S_U_*) dependencies on the forward current at 1 kHz frequency characteristic for group A and B LDs at room temperature.

Investigated devices can be classified into two groups—A and B—considering two different characteristic dependencies of the voltage fluctuation spectral density on the forward current. These experimental dependencies of samples are depicted in Figure 4. Thus, the group A LDs had two bumps (maxima) observed in the investigated current region, while group B had only one. The noise level of group B lasers was apparently higher (up to three orders of magnitude). The level of noise slightly varied (up to one order of magnitude) between LDs in the same group, but the number of observed bumps was constant.

**Figure 5:**
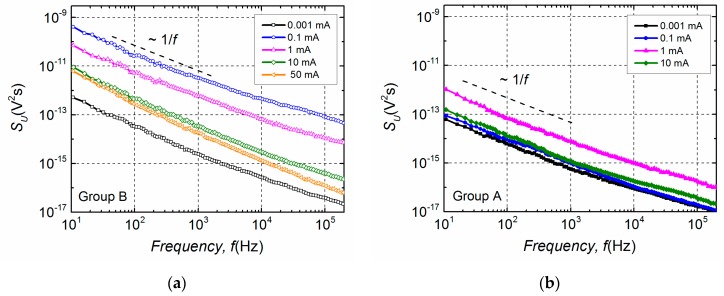
Electrical noise spectra *S_U_* of the group B LD (**a**) at different current values in the whole investigated forward current range and of the group A LD (**b**) at different forward current values up to the second bump appearance (presented in Figure 4).

**Figure 6:**
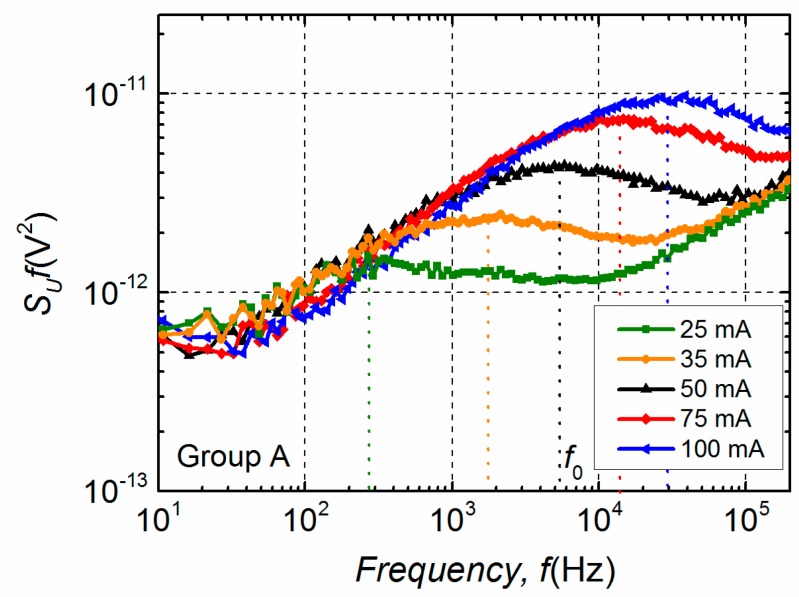
Normalized electrical noise spectra *S_U_ f* of the group A LD in the current range of the second bump (*I* > 15 mA in Figure 4).

**Figure 7:**
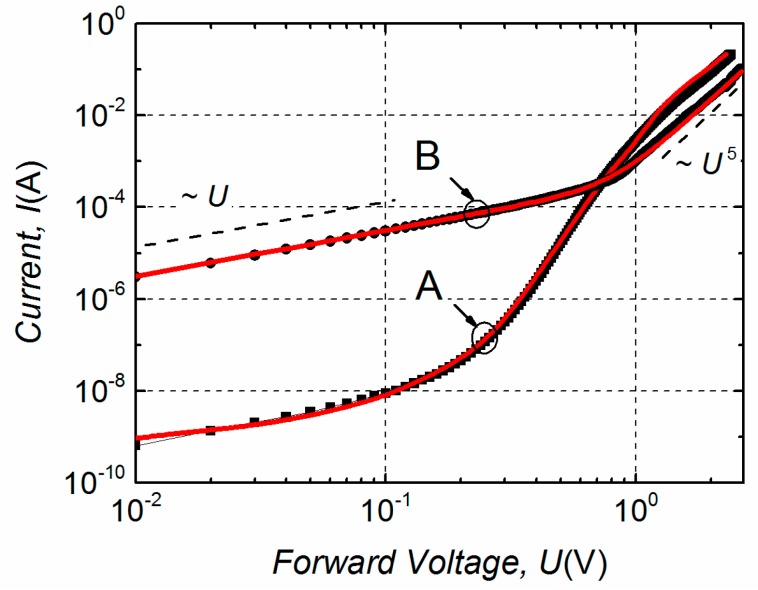
Typical *I*–*U* characteristics of the group A and B LDs in the current range far below the threshold; the black symbols represent experimental results and the red solid curves—an approximation by Equation (3) for the group A and by Equation (2) for the group B.

Furthermore, differences in electrical noise spectra are observed between these two groups. Low-frequency electrical fluctuations of the group B LDs are distinguished by 1/*f*^α^-type spectra in the entire investigated current region, where index α ranges from 0.9 to 1.4 (Figure 5a). In semiconductor devices, the 1/*f*^α^-type fluctuations originate from the superposition of Lorentzian-type spectra due to different charge carrier generation-recombination or capture-emission processes having wide distribution of relaxation times [19,20,21]. The electrical noise spectra of group A LDs, as it can be seen from Figure 5b, are also 1/*f*^α^-type in a forward current range up to the second bump. This second maximum starts in the current interval from ~15 mA to ~25 mA for different samples of group A (Figure 4). The appearance of the second bump is related to the change of noise spectra—a clear Lorentzian-type component is observed, which is characteristic for generation-recombination noise (Figure 6). There equation 2π*f*_0_*τ* = 1 holds for the maximum of the normalized g-r noise voltage spectral density with *τ* being the average relaxation time and *f*_0_—the characteristic frequency at the maximum of the spectrum. The origin of these g-r fluctuations will be discussed in the next Sections. As it could be expected, differences between electrical noise characteristics of the group A and B LDs are also reflected in their current–voltage characteristics (Figure 7).

### 3.2. Current–Voltage Characteristics of the Group A and B LDs

It is well known, that for the optoelectronic devices, such as light-emitting diodes (LEDs) or LDs, there is a strong relation between the electrical noise and current–voltage characteristics [17,22], e.g., leakage channel existence is usually followed by an increased low-frequency electrical fluctuations’ level [23].

Typical *I*–*U* characteristics of the investigated group A and B LDs are presented in Figure 7, where black curves with squares and dots represent the experimental data. A strong difference between these *I*–*U* curves is observed; the group B LD curve is far from an exponential dependence typical to an ideal diode and for the sake of convenience it can be written as a polynomial:

(2) $$I=aU+bU^{n};$$

where a and b are coefficients, and *n* is higher than 1 at forward voltages above 0.9 V. Meanwhile the *I*–*U* characteristic of the LD from group A can be expressed as usual:

(3) $$I=I_{0}\exp[q(U-IR_{s})/(\eta kT)]+a_{1}U;$$

where *I*_0_ is the saturation current of a diode, *q*—an elementary charge, *R*_s_—a series resistance, *η*—a non-ideality factor of the diode and the *a*_1_*U* term describes the additional current at low bias *U* (in Equation (3) exp[*q*(*U* − *IR*_s_)/(*ηkT*)] >> *I*_0_). To approximate these experimental dependencies (red curves in Figure 7) the index *n* = 5 in Equation (2) has been used and the non-ideality factor *η* in Equation (3) has been determined from the best fit with the experimental curve (*η* ≈ 2.5 in a current range from 10^−6^ A to 10^−4^ A; the other quantities in Equation (3) are *R*_s_ ≈ 3 Ω and *I*_0_ = 9.5 × 10^−10^ A).

### 3.3. Noise Equivalent Circuits of the LDs and Their Analysis

**Figure 8:**
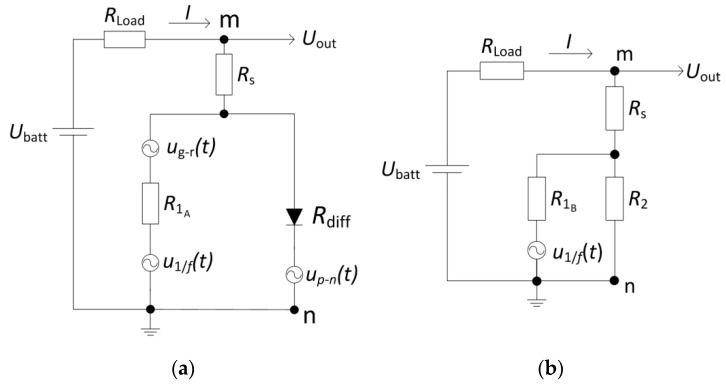
Equivalent electrical circuits of group A (**a**) and group B (**b**) LDs with the voltage noise sources (*R*_Load_ >> *R*_mn_).

Based on the results presented in Figure 4 and Figure 7, the equivalent electrical circuits of group A and B LDs are proposed in Figure 8. In these equivalent electrical circuits, the *R*_1_ describes parallel leakage resistance, however, it depends on applied bias differently for group A and B LDs. The diode symbol in Figure 8a represents an ideal *p*–*n* junction of the group A LDs with its own differential resistance *R*_diff_ = (d*U_p_*_–*n*_/d*I*). The corresponding differential resistance of group B LDs is denoted by *R*_2_ (Figure 8b) according to the non-exponential *I*–*U* characteristic approximation (Equation (2)). The *R*_s_ in Figure 8 represents the series resistance of contacts and highly doped *n*^+^ and *p*^+^ layers.

**Figure 9:**
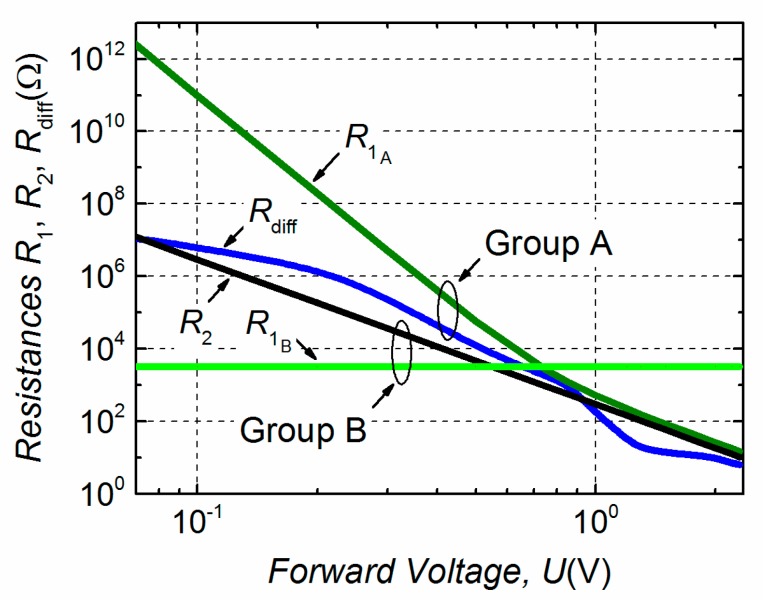
Calculated dependencies of *R*_1_, *R*_2_ and *R*_diff_ (presented in Figure 8) resistances on forward voltage.

Calculated dependencies of the discussed leakage and differential resistances on forward voltage are presented in Figure 9. Such graphical representation of these dependencies is very useful to explain the electrical noise properties. For the group B LDs, the linear part of the *I*–*U* characteristic determines the resistance *R*_1B_ (~3200 Ω). Then, by differentiating Equation (2) and eliminating the ohmic part, the resistance *R*_2_ can be expressed as *R*_2_ = 1/(0.0034 × *U*^4^). In the case of group A LDs, the dependence of the resistance *R*_1A_ on forward voltage has been evaluated regarding to the best coincidence between experimental data and approximated results of both *I*–*U* and electrical noise characteristics. *R*_1A_ decreases with increasing applied forward voltage more steeply in the bias range of the first electrical noise bump than in the second one (at *U* > 0.9 V) as it is shown in Figure 9.

For the 1/*f*-type electrical fluctuations the current noise spectral density, *S_I_*, for a *p*–*n* junction is proportional to the forward current [24]. As the flowing through the diode forward current increases, the differential resistance *R*_diff_ of an LD decreases and the voltage fluctuation spectral density is $S_{U}=S_{I}\cdot R_{\mathrm{diff}}^{2}$. The corresponding voltage noise source in the equivalent electrical circuit is denoted as *u_p_*_–*n*_(*t*) (Figure 8a). However, the influence of fluctuations originating from this noise source is visible only in group A LD (Figure 4) where the relation:

(4) $$S_{U\;p-n}=\frac{C}{f}\cdot \frac{1}{I^{\gamma }};$$

is up to *I* = 1 × 10^−5^ A and the index *γ* is close to 1.

For the group A LDs the proportionality *S_U_ ~ I^−γ^* is no longer valid when *I* > 1·10^−5^ A (Figure 4). As a result, there should be additional voltage noise sources located parallel to the *p*–*n* junction (Figure 8a). It was mentioned earlier that for the group A samples the type of electrical noise spectra changes in the forward current interval from ~15 mA to ~25 mA, where the Lorentzian-type fluctuations prevail 1/*f*-type. Thus, for *I* > 1 × 10^−5^ A the electrical noise spectral density *S_U_*_out_ can be expressed as a sum of two spectral densities of independent noise sources, *u*_1/*f*_ (*t*) and *u*_g-r_ (*t*):

(5) $$S_{U\;\mathrm{out}\;A}=S_{U\;1/f\;\mathrm{out}}+S_{U\;g-r\;\mathrm{out}};$$

where the 1/*f*-type fluctuations are calculated as:

(6) $$S_{U\;1/f\;\mathrm{out}}=\frac{DU^{2}}{f}\cdot {\left(\frac{R_{\mathrm{diff}}}{R_{1_{A}}+R_{\mathrm{diff}}}\right)}^{2};$$

and for the generation-recombination noise, i.e., the Lorentzian-type spectra fluctuations:

(7) $$S_{U\;g-r\;\mathrm{out}}=4EU^{2}\frac{\tau }{1+{\left(2\pi f\tau \right)}^{2}}\cdot {\left(\frac{R_{\mathrm{diff}}}{R_{1_{A}}+R_{\mathrm{diff}}}\right)}^{2}.$$

For group B samples, only the 1/*f*-type electrical fluctuations have been observed in a whole investigated current region and their spectral density can be described as:

(8) $$S_{U\;\mathrm{out}\;B}=\frac{FU^{2}}{f}\cdot {\left(\frac{R_{2}}{R_{1_{B}}+R_{2}}\right)}^{2}.$$

**Figure 10:**
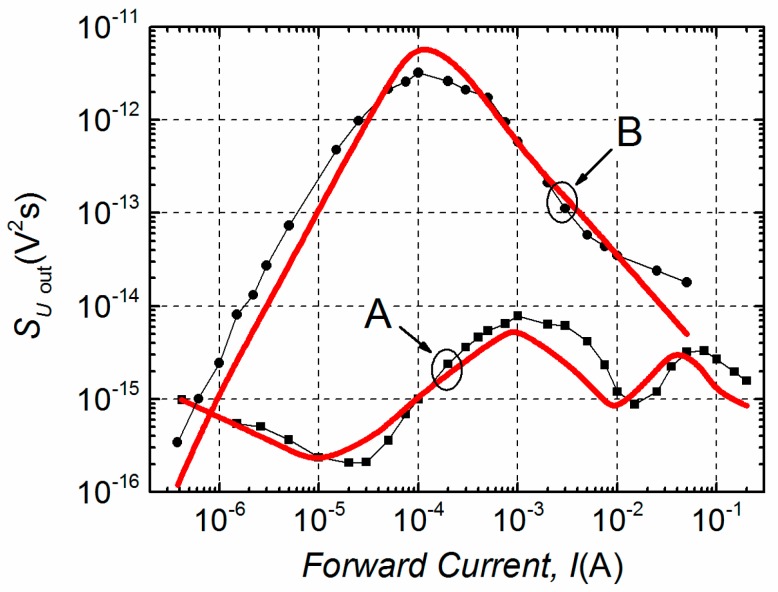
Comparison of the experimental voltage fluctuation spectral density dependencies on forward current at 1 kHz frequency (black curves with symbols) and the calculated data (red curves) for LDs of groups A and B at RT.

Here, in Equations (4), (6)–(8) C, D, E and F are coefficients. The comparison between experimental voltage fluctuation spectral densities and those calculated by Equations (4)–(8) are presented in Figure 10. The noise source *u_p_*_–*n*_(*t*) is not presented in Figure 8b because in the investigated current range the level of electrical fluctuations, arising from the *p*–*n* junction, is much lower than the level of fluctuations from the *u*_1/*f*_ (*t*) noise source.

### 3.4. The Origin of Electrical Fluctuations in GaAsBi LDs

The resistance *R*_1_ acts as a shunting resistance with respect to *R*_diff_ (or *R*_2_). For example, in a case of group B, the voltage fluctuation spectral density increases as *I*^2^ in a forward current interval up to ~0.1 mA (corresponding voltage ~0.5 V) (Figure 10), where *I ~ U* (Figure 7). At the bias values *U* > 0.5 V, the level of electrical fluctuation rapidly decreases as now *R*_2_ shunts *R*_1B_ (Figure 9). A similar electrical noise dependence on forward current with an evident one strong bump has been also observed in the GaSb-based laser diodes in a subthreshold region [17]. For GaAsBi group B LDs the leakage channel resistance *R*_1B_ is independent of the applied voltage (Figure 9) and the leakage current, corresponding to the ohmic part of the *I*–*U* characteristic, is larger up to four orders of magnitude compared to the samples from group A (Figure 7). This suggests that a surface leakage channel exists, which is responsible for the fluctuations observed in group B GaAsBi LDs. This channel is formed by the high density active g-r centers located on the LD surface that have widely distributed relaxation times leading to the 1/*f*-type electrical fluctuations spectra. The formation of capture centers on the surface is very sensitive to the quality of cleavage process of the LD when a periodic crystal potential breaks off at a semiconductor surface leading to the origin of surface states [25]. An appropriate surface passivation would decrease surface state density and, consequentially, could reduce the low-frequency noise and increase the threshold of a catastrophic optical damage [25,26,27].

The origin of the leakage resistance *R*_1_ in the group A LDs is different than in group B; the level of electrical fluctuations created by this resistance noise source is significantly lower (Figure 10) and *R*_1A_ depends on the applied voltage, i.e. it is not constant as *R*_1B_ (Figure 9). The voltage fluctuation spectral density of the first noise bump of the group A samples is increasing as *S_U_* ~ *I* up to *I* ≈ 1 mA (Figure 10). The second weaker maximum in the group A LDs’ electrical noise characteristic is related to the g-r noise appearance. The Lorentzian-type spectra emersion suggests that there is a strong active g-r center turning on at *U* > 0.9 V. For detection and identification of these local levels for the group A LDs and to relate them to the origin of the resistance *R*_1A_, the g-r noise spectroscopy has been applied.

**Figure 11:**
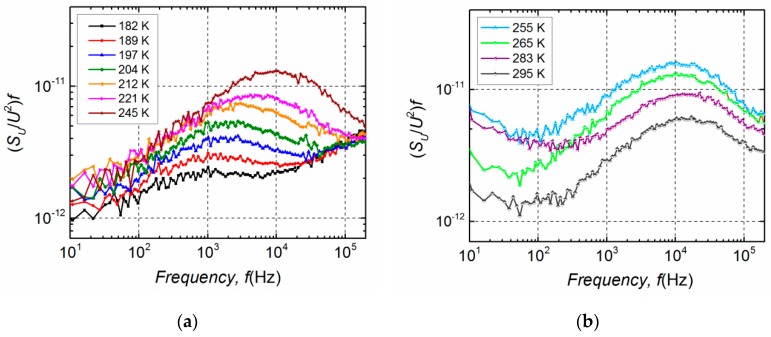
Normalized g-r noise spectra (*S_U_*/*U*^2^)*f* of the investigated group A LD at different temperatures at forward current *I* = 30 mA: in the temperature range from 182 K to 245 K (**a**) and from 255 K to the room temperature 295 K (**b**).

Normalized g-r noise spectra of group A LD at different temperatures are presented in Figure 11. When the temperatures are below 250 K (Figure 11a) the relaxation time *τ* = 1/(2π*f*_0_) is dependent on temperature and is described as: $\tau =\tau_{0}\exp(E_{a}/kT)$ [17], where *E*_a_ is the activation energy of an observed g-r process. The maximum (*S_U_*/*U*^2^)*f* value appears when the Fermi level coincides with the trap energy level [13,28]. At temperatures *T* > 250 K the relaxation time is almost independent of temperature showing that the Fermi level is bound to the accumulation of defect levels in a particular place of the bandgap of the LD active region (Figure 11b).

**Figure 12:**
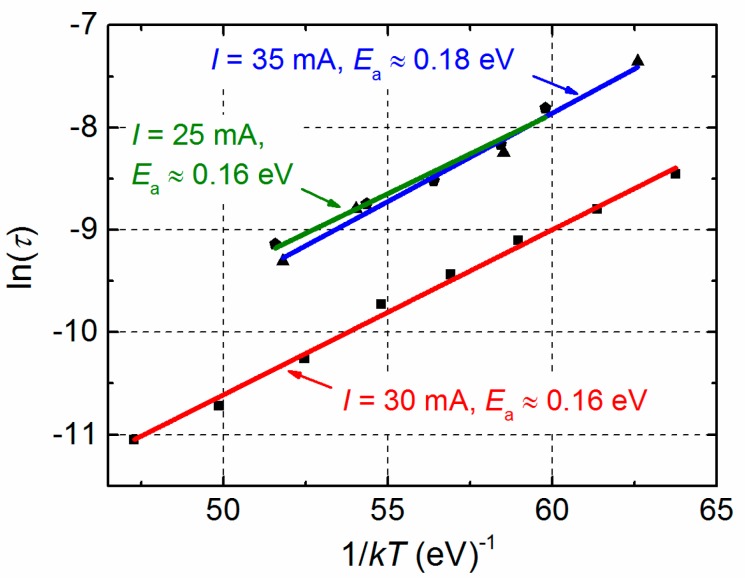
Dependencies of the relaxation time on the inverse temperature of different group A samples. The evaluated activation energies *E*_a_ and experimental conditions are presented near the curves (square symbols denote ln (*τ*) values obtained from LD electrical noise spectra presented in Figure 11a).

The activation energies of the corresponding relaxation processes of different group A LDs’ have been found in the range from 0.16 eV to 0.18 eV (Figure 12). Similar activation energies have been observed in MBE grown *n*-type GaAs_1-x_Bi_x_ layers on GaAs substrate by deep level transient spectroscopy (DLTS) [9]. In [9], it has been identified that the defects involving Bi atoms in their structure are responsible for detected trap levels. It is known that Bi incorporation into GaAs at low temperature enhances Bi surface migration, Bi cluster formation [3,4,8] and at the same time Bi-related defects are present [9]. According to the evaluated activation energy, the influence of strong g-r center created by defects involving Bi is increasing at *U* > 0.9 V leading to the Lorentzian-type fluctuations in electrical noise spectra. So, the second noise bump of the group A LD (Figure 10) is related to the LD active multi-quantum well (MQW) region (where GaAsBi QWs are embedded and its interfaces). While the first bump in the group A LD voltage fluctuation spectral density with characteristic 1/*f*^α^-type electrical fluctuations is determined by sample defects and various charge carriers capture or release centers in the active region as well as in peripheral areas of the LD.

## 4. Conclusions

Low-frequency noise characteristics of 1.09 μm GaAsBi laser diodes have been investigated in the current range far below lasing threshold. Considering different voltage fluctuation spectral density dependencies on the forward current, the investigated samples have been classified into two groups; A and B.

Laser diodes of the group A are distinguished by two bumps in the electrical noise dependence on current. The first one is characterized by 1/*f*^α^-type electrical fluctuations, the second one by Lorentzian type spectra with the activation energy (0.16–0.18) eV. The analysis of the current–voltage characteristics and the equivalent noise circuits has shown, that the origin of the first electrical noise bump is determined by the overall LDs (the active and the peripheral areas) defectiveness, while the second one by the large density g-r centers due to Bi-related defect states in the active MQW LDs layer and its interfaces.

The group B LDs are distinguished by one steep electrical noise bump in the dependence on forward current with 1/*f*^α^-type electrical fluctuations in the whole investigated current range. In this case, equivalent circuit analysis has shown that the leakage resistance is independent of applied bias and the corresponding ohmic part of *I*–*U* characteristic is much higher compared to the group A LDs. The origin of these fluctuations is the leakage channel formed at a surface of LDs due to high density surface defects and charge carrier capture centers.
